# Department of Practice of Medicine

**Published:** 1893-03

**Authors:** Allen J. Smith

**Affiliations:** Medical Department University of Texas, Galveston


### DEPARTMENT OF PRACTICE OF MEDICINE.

Conducted by Prof. Allen J. Smith, A. M., M. D., Medical Department University of Texas, Galveston.

MENTAL SYMPTOMS OF DIABETES.—M. J. Madigan, of Brooklyn (*Medical Standard*, February, 1893), reports several instances of the occurrence of glycosuria and insanity in the same individual. One of the common manifestations of glycosuric insanity is an alternation of the diabetic and mental phenomena; and a case reported by the writer well illustrates it. A thirty-year-old Scottish-American was admitted to hospital with decided emotional exaltation, grandiose delusions, a kleptomaniac, destructive, and occasionally violent. No albumen or sugar was present in his urine during the period of these symptoms. After a time he became quieter, the expansive ideas less prominent, the moral obliquities which he had manifested disappeared, and gradually the man passed into mental convalescence and on to recovery. At the beginning of mental improvement, traces of sugar appeared in the urine, and gradually increased as the case progressed toward recovery. At the same time his appetite increased. In four months he was discharged, apparently cured of his insanity, but was distinctly diabetic. In the spring of the following year he again came under care in much the same mental state as at first. Sugar was not to be detected in the urine on admission, nor during the period of mental disturbance. When convalescence came on, sugar again made it arrearance. After discharge the second time, the patient remained away from the hospital for a long time, but eventually returned to die of diabetic phthisis. The mental symptoms of the attack in which he died were of a melancholic type. Just before this last attack he had been placed under great mental strain, and had in addition caught a severe cold.

---

ALBUMEN REACTION-RESEMBLANCE OF PIPERAZINE. — H. Berlin (*Virginia Med. Monthly*, February, 1893), of Chattanooga, calls attention to an albumen-like reaction occurring in the urine of patients taking piperazine, upon the addition of picric acid solution. The reaction fails with nitric acid and the other common albumen reagents.

---

THE CONDITIONS OF HIGHER MEDICAL EDUCATION.—The wretched condition of medical education in our country has long enough been the subject of ridicule of other countries, and of the best men among ourselves. It is a shame that the whole profession in the United Stated should set itself rigorously and vigorously to stop. State boards of examination are right, and should be established everywhere; but they are not sufficient. These can only properly raise their standards as the applicants for admission have been better prepared by the colleges.

About the following propositions it would appear there can be no possibility of discussion or doubt:

1. The minimum of study requisite for granting the degree of M. D. to students possessing no literary or scientific training upon entering upon the medical studies, should either be four years, or three years of lengthened terms, with real and not sham entrance examinations, and with a teaching and drilling in actual medical and clinical work that is adequate, and is not merely commercialism masquerading as medicine. Three years —such years as are usually passed—have become certainly and plainly insufficient.

2. No institution can adequately fit its students for the work without endowment or State aid. As a rule, the colleges possessing endowment will certainly prepare their students better than those without the necessary laboratories, the large corps of paid assistants, the enlarged teaching body, the drill of bedside instruction, etc.

3. The fixation of salaries of the teaching body is an absolute prerequisite to elevation of standards, and a prevention of the graduation of the ill-fitted. It is also a prerequisite for obtaining endowment, as neither individuals nor the community should endow or aid an institution whose profits are for the benefit of individuals, and only secondarily and adventitiously of medical education and of the profession.—*Medical News*, February 4, 1893.

---

THE TREATMENT OF CHOLERA.—Professor Roberts Bartholow (*Med. News*, February 4, 1893), in a recent article reviewing the treatment of cholera, outlines the theory of a cholera seizure as follows: The parasite reaching the intestine by some one of the channels of communication, lodges in the mucous membrane and there multiplies, producing at the same time a toxic principle. As a result of the irritation arising from the presence of the parasite and of the toxine, there occurs as an early symptom the preliminary diarrhœal stage; this is followed by a stage of systemic infection due to the absorption of the toxine, quickly succeeded by the algid stage as the result of the special effect of the latter, and then, if reaction occur, by a typhoid condition. At the same time, severe desquamation of the mucous membrane goes on; and serious effusion from the blood vessels takes place to such an extent as to thicken the blood and alter the power of functionation of the red blood cells.

Professor Bartholow holds forth as the first indication the arrest of the diarrhœa, guiding his selection of remedies by the recollection of the presence in the intestines of a parasite, of the excessive alkalinity of the fluids and tissues, and of the effusion of the blood serum into the intestine. Of all remedies suggested he regards, from his own experience, as the best, sulphuric acid, and suggests its administration as follows:

℞ Acidi sulphurici aromatici . . . . . . . . . . . ʒ v

Tincturæ opii deodoratæ . . . . . . . . . . . ʒ iij

M. Sig.: Gtt. x–xx every hour or two, in sufficient water.

Or:

℞ Acidi sulphurici diluti . . . . . . . . . . . . . ʒ iij

Tincturæ opii camphoratæ . . . . . . . . . ʒ xiij

M. Sig.: Teaspoonful every half hour, or hour, in water.

The writer counsels that opium be not removed from the list of proper remedies, although he acknowledges the propriety of small dosage of the drug, rather than the heroic doses given formerly.

The discovery of the comma bacillus has led to the employment of various germicidal remedies, and salol and creolin have been viewed with high expectation. Generally speaking these expectations have not been realized. The author is inclined to believe that better results may be obtained from naphthalin, and suggests its combination with bismuth and carbolic acid, as follows:

| | | |
|---|---|---|
| ℞ | Naphthalin | ʒ ss |
| | Bismuthi subnitratis | ʒ ij |
| | Acidi carbolici | ʒ ss |
| | Glycerini | ℥ ss |
| | Aquæ chloroformi | ℥ iss |

M. Sig.: Teaspoonful every half hour, hour, or two hours.

Excellent results seem to be attending the employment of calomel in the present epidemic, as was the general experience also in previous ones, the remedy being given at first in a single large dose followed by smaller doses at short intervals thereafter.

*Enteroclysis*, the irrigation of the intestine with a warm solution (99°–104° F.) of tannic acid (tannic acid, dr. iss-iiss-v, water Oii-iv, laudanum gtt. xx-xxx, gum arabic oz. j-iss), is mentioned as a procedure of little value, in spite of the earnest advocacy of Cantani, Ziemssen, and others. The writer does not believe the injection of a fluid into the large intestine is followed by its entrance into the diseased ileum, and can thus modify a morbid process in the latter situation. Intra-venous and hypodermatic (*hypodermatoclysis*) infusion of a saline solution, usually one containing sodium chloride or sodiun carbonate, have been practiced extensively during the present epidemic, although the writer credits little but temporary benefit to either method. The relatively safer and easier employment of the latter mode of administration has rendered it preferable to most practitioners in private work, although no definite conclusion as to the proportionate value of the two methods has been reached. In the algid state, with fatal collapse imminent, Professor Bartholow suggests, however, that the intra-venous infusion is preferable, as productive of a more rapid result.

The writer also refers to anti-cholerine, given in subcutaneous injections, the mortality from this mode of treatment being lower, or at least not higher, than that from other methods. The substance, anti-cholerine, is derived from pure cultures of the cholera spirilla by Klebs, in the same manner Koch derives tuberculin from cultures of tubercle bacilli.

The treatment during the algid stage in the present epidemic has not varied much from that of previous ones. The soluble salts of caffeine, given for their diuretic effect and the purpose of circulatory stimulus, have achieved some reputation in Paris; as, too, has cocaine, injected subcutaneously to the amount of half a grain per day. Nitroglycerine (gtt. ij of a one per cent. solution on the tongue) and the hypodermatic use of ammonia, have also been recommended. Bartholow suggests that, in view of the desquamated condition of the intestinal mucous membrane, the nitroglycerine, or nitrate of amyl, had best be administered hypodermatically; or amyl nitrate may be given by inhalation. These remedies had best be given after the injection of the saline solution, since there must be little use of circulatory stimulus while the blood is thickened from loss of its serum. For the cramps occurring at this stage, small doses of morphine and chloral are recommended, and Bartholow suggests the following as a suitable formula for hypodermic administration:

℞ Chloral hydratis . . . . . . . . . . . . . . ʒ iij

Morphiæ sulphatis . . . . . . . . . . . . . gr. j

Atropinæ sulphatis . . . . . . . . . . . . . gr. ¼

Aquæ chloriformi,

Aquæ . . . . . . . . . . . . . . . . . āā ℥ss

M. Sig.: Twenty minims, repeated in ten minutes, and subsequently *pro re nata.*

As to diet, the author urges that when the intestinal tract is undergoing desquamation, attempts to feed are worse than useless. Such a preparation as beef-tea is only harmful at any stage of the disease. A little cold milk or wine whey may be given as the stomach permits. The tendency to overuse of stimulants is very strong, and care should be had lest this mistake be fallen into. Small quantities of iced champagne or iced brandy may be given from time to time. Although excessive drinking is apt to provoke or renew vomiting, the advantages from free use of water are thought to more than counterbalance the injury thus aroused. Carbonic acid water and such a mineral water as Apollinaris may be less likely to disturb the stomach; and the restoration of the renal function and the diluent action of the water are undoubtedly elements of importance in the scheme of treatment.

